# Molecular Rotors: Fluorescent Sensors for Microviscosity and Conformation of Biomolecules

**DOI:** 10.1002/anie.202311233

**Published:** 2023-11-14

**Authors:** Miguel Paez‐Perez, Marina K. Kuimova

**Affiliations:** ^1^ Department of Chemistry, Imperial College London, MSRH Imperial College London Wood Lane London W12 0BZ UK

**Keywords:** Biophysics, FLIM, Lipid Membranes, Microviscosity, Molecular Rotors

## Abstract

The viscosity and crowding of biological environment are considered vital for the correct cellular function, and alterations in these parameters are known to underly a number of pathologies including diabetes, malaria, cancer and neurodegenerative diseases, to name a few. Over the last decades, fluorescent molecular probes termed molecular rotors proved extremely useful for exploring viscosity, crowding, and underlying molecular interactions in biologically relevant settings. In this review, we will discuss the basic principles underpinning the functionality of these probes and will review advances in their use as sensors for lipid order, protein crowding and conformation, temperature and non‐canonical nucleic acid structures in live cells and other relevant biological settings.

## Graphical abstract

In this review, we describe recent advances in the development of fluorescence‐based viscosity sensors, termed *Molecular Rotors*, and discuss how these dyes can be exploited to quantify the microviscosity of biological environment, probe lipid membranes and non‐canonical nucleic acid architectures and track dynamic processes, including protein aggregation.

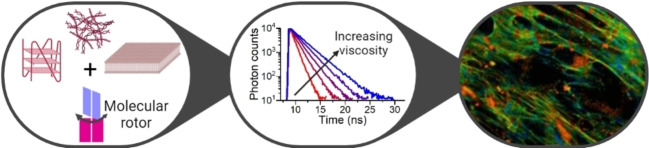

## 1 Introduction

Viscosity, which is the inverse parameter of fluidity, reflects a material's resistance to deformation for a given deformation rate. It could be defined as a proportionality coefficient between the sheer stress, τ, and a velocity gradient, ∂u/∂y, caused by a force acting on the top‐most layer of an object:

(1) $$\tau =\;\eta \frac{\partial u}{\partial y}$$

where η is known as the dynamic viscosity and has units of Pa s.

Unsurprisingly, material viscosity plays a key role in biology across several length scales.^[1]^ For example, the cartilage's viscoelastic properties affect osteoarthritis progression,^[2]^ while the extracellular matrix viscosity is thought to influence mechanotransduction^[3]^ and cancer dissemination.^[4]^ There is mounting evidence that the fluidity of the cell's lipid plasma membrane is tightly regulated to enable the optimal cellular function,^[5]^ and viscosity within the cell's nucleus may influence the formation of non‐canonical DNA assemblies, such as G‐quadruplexes.^[6]^ However, unlike in the cases of bulk industrial applications including additive manufacturing,^[7]^ polymerization,^[8]^ or food processing,^[9]^ biological viscosity is challenging to measure and requires the development of methodologies that are able to probe viscoelastic properties of microscopic volumes of individual biological cells and organelles.

On one hand, deformation‐based approaches, such as microfluidic setups,^[10]^ micropipette aspiration,^[11]^ electro‐deformation,^[12]^ optical and/or magnetic traps,^[13]^ or high‐speed AFM indentation,^[14]^ make use of force to deform the material, and the viscosity values can be extracted by monitoring the responses. However, in biological environments the use of these approaches is often prohibited by their need for a non‐native environment, their inability to simultaneously probe multiple positions, and/or low measurement throughput. On the other hand, spectroscopic techniques including fluorescence correlation spectroscopy (FCS),^[15]^ fluorescence recovery after photobleaching (FRAP),^[16]^ or single particle tracking (SPT)^[17]^ are based on tracking the diffusion of a fluorescent label as a proxy for measurement of the viscosity of the surrounding microenvironment. Finally, environmentally sensitive fluorescent probes can be utilized to map crowding and viscosity in their local microenvironment.^[18]^

In this review we will discuss the working principles and the applications of a class of environmentally sensitive dyes termed “molecular rotors”, in which fluorescence emission parameters are viscosity‐dependent. We will particularly focus on measuring viscosity and crowding in soft matter assemblies of biological relevance, including nucleic acids, proteins, and lipid membranes. We will discuss the roles of temperature and macromolecular conformation of biomolecules as confounding variables in these measurements.

## 2 Theoretical background

### 2.1 Working principle of molecular rotors

The discovery of molecular rotor‐like function dates back to 1980, when *Law at al*. demonstrated that a donor‐acceptor fluorescent malonotitrile derivatives exhibited a higher fluorescence intensity when dissolved in solvents of increasing viscosity.^[24]^ This behaviour was attributed to the competition between non‐radiative and radiative decay pathways. Return to the ground state without fluorescence emission was favoured in the presence of efficient intramolecular rotation, which was less likely to occur in a more viscous media. Therefore, the higher viscosity led to a higher fluorescence intensity.

**Figure 1:**
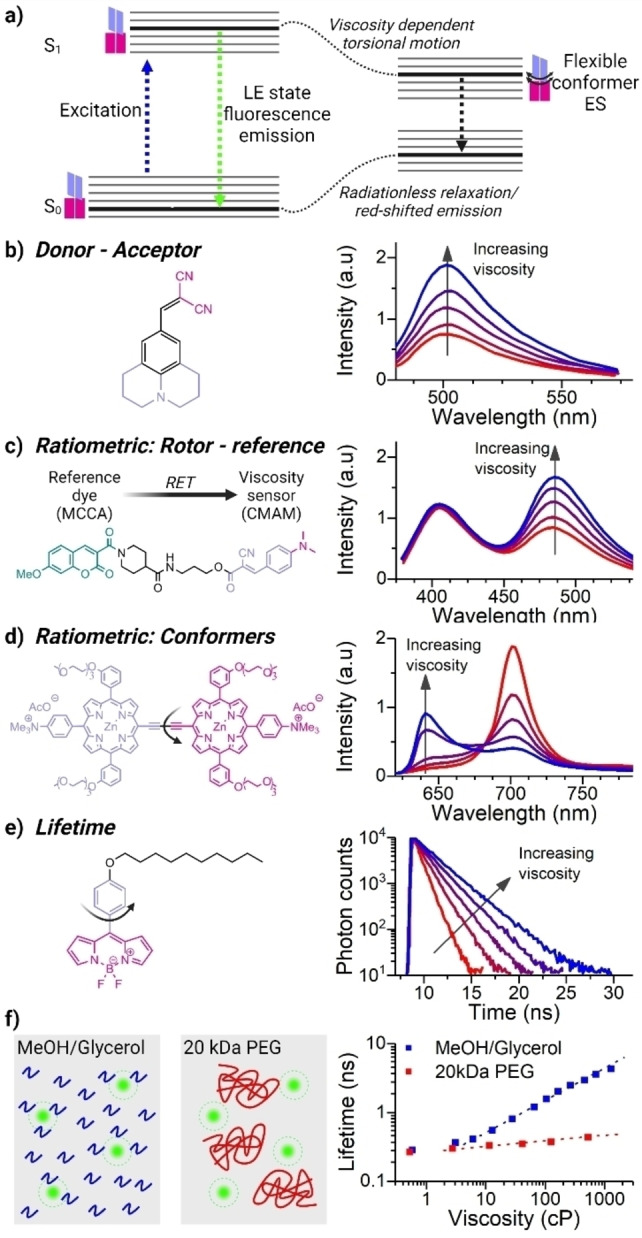
Molecular rotors are fluorescence‐based viscosity probes. a) Jablonski diagram showing the generic working principle of molecular rotors: excitation to a locally excited (LE) state is followed either by the radiative decay or by twisting, the competition between these two pathways is affected by the viscosity of the rotor's environment. b–e) Examples of molecular rotors based on different readouts; b) dark twisted intramolecular charge transfer (TICT) state formation in a donor‐acceptor system (Adapted with permission from ref. [19] Copyright 2010, Springer‐Verlag Berlin Heidelberg), c) spectral ratiometric rotors, where only one of the linked fluorophores is viscosity‐dependent (Adapted with permission from ref. [20] Copyright 2006, American Chemical Society), d) viscosity‐dependent conformer‐based spectral ratiometric rotor where both conformers are emissive (Adapted with permission from ref. [21] Copyright 2017, Wiley‐VCH GmbH) and e) fluorescence lifetime‐based rotor (Adapted with permission from ref. [22] Copyright 2013, National Academy of Science). f) The effect of solvent free volume on the rotor's readout: true molecular rotors (in green) sense micro‐viscosity, which increases upon the reduction in solvent free volume at increased concentrations of small solutes (blue),^[22]^ whereas dilute solutions of large macromolecules do not affect the signal (red).^[23]^ (Adapted with permission from ref. [22] Copyright 2013, National Academy of Science and ref. [23] Copyright 2020, Royal Society of Chemistry).

While the first reported molecular rotors were generally constructed from three subunits: an electron donor, an electron acceptor and an electron‐rich spacer unit, which brings the donor and acceptor in conjugation,^[18b]^ it became apparent that the resulting excited state charge separation that is promoted in the non‐viscous solvents also creates artefacts. Polarity depedence of the emission can occur, in addition to the desired viscosity response, due to the charge stabilisation of the twisted intramolecular charge transfer excited state (TICT), and even unwanted spectral shifts.^[25]^ However, more recently, a variety of molecular rotor structures were proposed, that rely on the dark state population due to intramolecular geometry change, where TICT does not contribute artefacts,^[26]^ or rotors with no TICT, and even rotors in which both excited states affected by intramolecular twisting are emissive.^[18a]^ The generalised energy diagram demonstrating the interrelationship between the native and the twisted excited states of various reporter rotor molecules is shown in Figure 1. Following excitation to S_1_, the rotors could proceed along two decay pathways. If intramolecular torsional motion is not restricted (e.g. in a low viscosity environment), thermal energy is sufficient to overcome the viscosity‐dependent energy barrier towards a more flexible excited state, that can be dominated by quenching (Figure 1b,c,e) or be emissive at a different wavelength (Figure 1d). On the contrary, when intramolecular motion is restricted (e.g. due to high viscosity) the rotor remains in the locally excited S_1_ state, allowing LE fluorescence. Similar torsional motion‐fluorescence quenching relationship also underlies the Aggregation‐Induced Emission (AIE) effect.^[27]^

Altogether, this implies that, for all these structures, drastic changes in the photophysical properties of molecular rotors, such as fluorescence quantum yield or lifetime, connected to an increase in the non‐radiative rate in a non‐viscous environment, can be used as a measure of local viscosity.

The relationship between the photophysical parameters of molecular rotors and the solvent's viscosity η was quantitatively described by Förster and Hoffmann in 1971:^[28]^

(2) $$\log_{10}F=c_{1}+c_{2}\log_{10}\eta$$

Where F represents a fluorescence descriptor (such as quantum yield, QY; or lifetime, τ ) and $c_{i}$ represent calibration constants. This relationship allows to establish the responses of the molecular rotor by measuring the QY or lifetime in a series of homogeneous solutions of known bulk viscosity, e.g. polar methanol/glycerol, water/sucrose mixtures, or non‐polar toluene/castor oil.[^18a^, ^29^]

It is important to mention that fluorescence properties of molecular rotors are affected by a very local *microscopic* viscosity, which is well described by a concept of the solvent free volume, rather than a bulk *macroscopic* viscosity featured in Eq 1. The theoretical framework to account for this effect was described by *Loutfy* et al.^[24b]^ Thus, macroscopically viscous solutions of *dilute* macromolecules (polymers,[^23^, ^30^] biomolecules^[31]^) should not result in high micro‐viscosity readouts by the rotors, Figure 1f. However, concentrated and crowded solutions of macromolecules with restricted solvent free volume will result in high rotor readouts (see below, Figure 5).

### 2.2 Quantitative measurements of viscosity using molecular rotors

The early viscosity measurements using molecular rotors were accomplished by measuring the QY and/or the fluorescence intensity of fluorophores, according to Eq. 2. However, since intensity‐based measurements are affected by the fluorophore concentration, they can only be converted to absolute viscosity values when the concentration of the fluorophore is known, which is rarely the case in microscopically‐heterogeneous samples of interest in biology. Thus, in samples such as cellular organelles, it is often only possible to detect the relative changes in viscosity upon the action of a stimulant, and only under an assumption that the concentration of the fluorophore in the region of interest does not change. Julolidine‐based rotors (Figure 1b) were used by *Haidekker* and co‐workers to explore the effect of shear stress^[32]^ or the addition of alcohols^[33]^ on the viscosity of artificial and cellular lipid membranes. Other elegant uses of intensity‐based molecular rotors include the study of binding interactions between proteins in vitro,^[34]^ visualization of flow patterns,^[35]^ tracking viscosity changes in polymer films,^[36]^ sensing contacts between surfaces,^[37]^ following gelation processes,^[38]^ the effect of altered gravity on cultured cells^[39]^ or measurements of red blood cells rheology,^[40]^ among others. However, the results of such intensity‐based measurements should be interpreted with caution, due to uncertainties of the probe's concentration, variations in the optical properties of the medium studied, and can be additionally affected by photobleaching.

One way of overcoming this challenge is through the use of spectral ratiometric rotors, Figure 1c,d. This approach exploits the ratiometric viscosity‐dependent signal, which is concentration‐independent. The ratiometric method of detecting viscosity was first reported by *Luby‐Phelps* et al. in 1993, who used a mixture of viscosity‐sensitive Cy3 and a reference Cy5 dyes, to measure the cellular cytoplasmic viscosity.^[41]^ However, by using non‐conjugated dyes, their co‐location and identical uptake could not be guaranteed. To address this limitation, *Haidekker* et al. developed a ratiometric molecular rotor composed of a 2‐*c*yano‐3‐(4‐di*m*ethylaminophenyl)*a*crylic acid *m*ethyl ester CMAM viscosity sensitive unit covalently linked to a 7‐methoxycoumarin‐3‐carboxylic acid moiety, used as an internal reference (Figure 1c).^[20]^ In this case, the viscosity‐insensitive unit allowed to perform ratiometric measurements of viscosity of lipid bilayers.^[42]^ This work was followed by others and resulted in further dye developement for measuring the viscosity in mitochondria^[43]^ or in whole HeLa cells.^[44]^

An alternative design for ratiometric rotors involves complex molecules that exist as viscosity‐dependent conformers, each characterised by a distinct emission band, e.g. conjugated porphyrin dimers (Figure 1d).[^21^, ^45^] In these dimers, the planar and the twisted conformers, defined with respect to the porphyrin monomers’ relative orientation, are characterised by very distinct emission bands, due to a different degree of conjugation present in each. We found that the interconversion between these forms, and, as a result, the ratiometric emission response of the dimers, strongly depends on the environmental viscosity. We utilised the ratiometric dimer sensors to measure the microviscosity of model and cellular membranes, as well as monitor changes upon photo‐oxidation.[^45a^, ^46^] Cyanine dyes with modified bridging groups were also found to produce ratiometric viscosity‐dependent responses, that were used to monitor mitochondrial viscosity.^[47]^

A very popular technique to monitor microscopic viscosity independently of the rotor's concentration is Fluorescence Lifetime Imaging microscopy (FLIM), pioneered by *Kuimova* et al. in 2008.^[48]^ In these measurements, the time‐resolved fluorescence decay of the rotor is measured as a function of viscosity, resulting in viscosity‐dependent fluorescence lifetimes (Figure 1e). Lifetime measurements provide significant advantages, since they are independent of the concentration of the probe, at least in the absence of aggregation and self‐quenching. FLIM measurements are also free from artefacts associated with the optical setup and transmission properties of the measured medium, which are more likely to affect spectrally‐disperse intensity‐based ratiometric measurements. However, while the time‐domain lifetime measurements are very accurate and mostly artefacts‐free, they require more specialised instrumentation and are significantly slower compared to a ratiometric data aquisition, due to the need to collect individual photons via time‐correlated single photon counting.

One important aspect to consider when using viscosity‐sensitive probes for quantitative measurements, performed either by lifetime‐based or ratiometric responses, is the possibility of multiple, perhaps interconnected, physical properties of the environment—such as pH,^[49]^ polarity,[^29a^, ^50^] or temperature[^29a^, ^51^]—influencing the readout of the rotors. For example, fluorophores sensitive to both pH and viscosity were previously described.^[52]^ The probing of multiple parameters can, however, be justified, if their measurements can be decoupled. For example,*Yu* and co‐workers designed a morpholino‐substituted BODIPY rotor in which the lower pH reduced intramolecular Photoelectron Transfer (PeT) and, hence, switched on the fluorescence without affecting the rotor's lifetime response to viscosity.^[53]^ Likewise, *Robson* et al.^[54]^ designed a turn‐on dual carbon monoxide (CO) and viscosity sensor. Here, BODIPY rotor's fluorescence was activated via a trans‐effect on the Ruthenium centre, caused by an increase in the concentration of free CO and its consecutive binding to Ru. The fluorescence lifetime of the rotor was responsive to viscosity alone, thus allowing the measurements of both parameters: viscosity (via lifetime) and CO concentration (via fluorescence intensity), with both parameters important in cellular inflammation. We have also investigated the applications of rotors to measure temperature, or temperature and viscosity simultaneously, which is described in detail below in the *Measuring Temperature using molecular rotors* section.

However, in some cases the changes in several environmental parameters can not be decoupled (e.g. viscosity and temperature,[^29a^, ^55^] or viscosity and polarity[^29a^, ^55b^]) and this means that the data from such probes should be interpreted with caution. Accurate data can still be obtained by fixing a second interfering variable (e.g. by performing imaging at a fixed temperature, or by ensuring that the calibration mixture of lifetime *vs* viscosity has the same polarity as an environment of interest).

Given the known advantages of both the lifetime‐based and spectral ratiometric‐based molecular rotor probes, several rotors were designed to combine both readouts: lifetime and ratiometric.[^46b^, ^56^] The dual‐mode response of such probes allows an independent verification of the results of either method and can reveal an unexpected sensitivity to confounding environmental factors, such as temperture, polarity or aggregation. In some cases, the dual response detection even allowed additional unique capabilities, such as monitoring of viscosity and temperature simultaneously.^[46b]^

**Figure 2:**
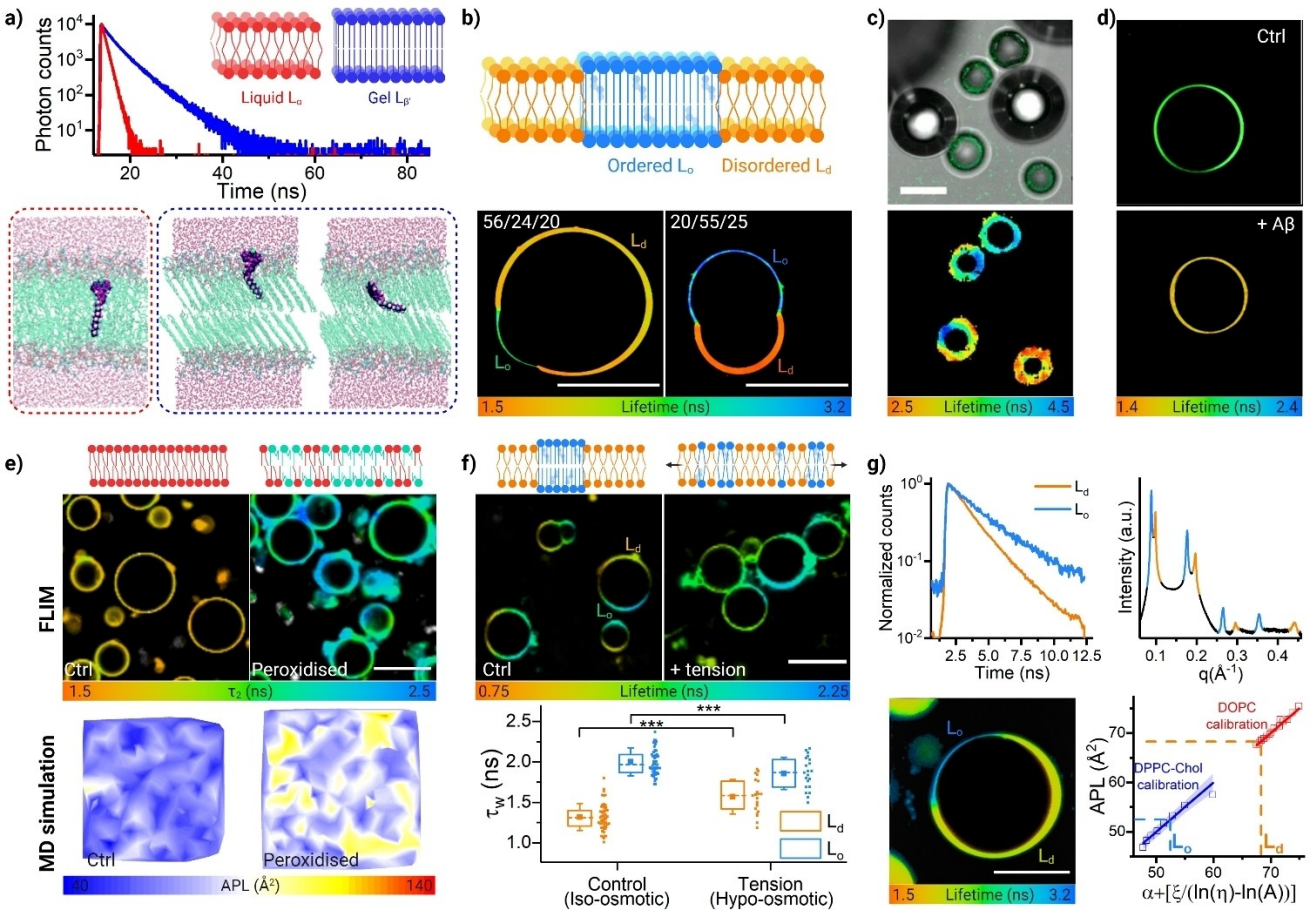
BODIPY‐based molecular rotors and FLIM imaging enable the quantitative mapping of the lipid membrane's microviscosity. a) MD simulations showing the rotor's single location in fluid phase lipid bilayers (L_α_, shown in red) and the two expected locations in tightly packed gel‐phase (L_β_, in blue) membranes. Accordingly, a single‐lifetime decay is observed in fluid lipid phases and a biexponential decay is observed in gel phases. (Adapted with permission from ref. [57a] Copyright 2015, Royal Society of Chemistry) b) FLIM imaging of phase‐separated GUVs of different composition (1,2‐Dioleoyl‐sn‐glycero‐3‐phosphocholine/egg yolk sphingomyelin/Cholesterol, DOPC/EYSM/Chol, the composition is shown above each image), in which liquid ordered (L_o_) phase has a significantly higher viscosity compared to the liquid disordered (L_d_) phase. Monoexponential decays are observed in both phases, and higher percentage of EYSP/Chol results in higher viscosity in both phases.^[57a]^ Scalebar: 20 μm. (Adapted with permission from ref. [57a] Copyright 2015, Royal Society of Chemistry) c) FLIM imaging of lipid‐coated microbubbles, revealing increased viscosity at the points of inter‐bubble contacts. Scalebar: 20 μm. (Adapted with permission from ref. [22] Copyright 2013, National Academy of Science) d) Effect of amyloid β (Aβ) oligomers on membrane viscosity of HeLa‐derived plasma membrane derived lipid vesicles. Scalebar: 30 μm. (Adapted with permission from ref. [68] Copyright 2018, Royal Society of Chemistry) e) Lipid peroxidation induces increased membrane viscosity monitored by FLIM (top) and MD simulations (bottom). The data for control GUVs (1‐palmitoyl‐2‐oleoyl‐glycero‐3‐phosphocholine, POPC) are shown as compared to peroxidised sample (synthetic POPC‐OOH); the formation of rigid lipid clusters is seen in FLIM and was confirmed by MD simulations. Scalebar: 30 μm. (Adapted with permission from ref. [57c] Copyright 2023, Springer Nature) f) FLIM imaging with thiophene rotors monitors tension‐induced changes in membrane viscosity: tension buffering is observed in phase‐separated GUVs (DOPC/DPPC/Cholesterol, 40/40/20, DPPC=1,2‐dipalmitoyl‐sn‐glycero‐3‐phosphocholine), in which L_o_ and L_d_ phases become closer in viscosity upon hypo‐osmotic tension, as measured by FLIM. Scalebar: 30 μm. (Adapted with permission from ref. [55b] Copyright 2020, Royal Society of Chemistry) g) Rotor lifetime measured in phase‐separated GUVs (left) can be directly correlated to X‐Ray derived Area‐per‐lipid parameter measured in bulk samples (APL, right). It could be seen that the calibration is specific to individual lipid phases (L_o_ in DPPC/Chol shown in blue and L_d_ in DOPC shown in red). Scalebar: 30 μm. (Adapted with permission from ref. [82] Copyright 2023, American Chemical Society).

**Figure 3:**
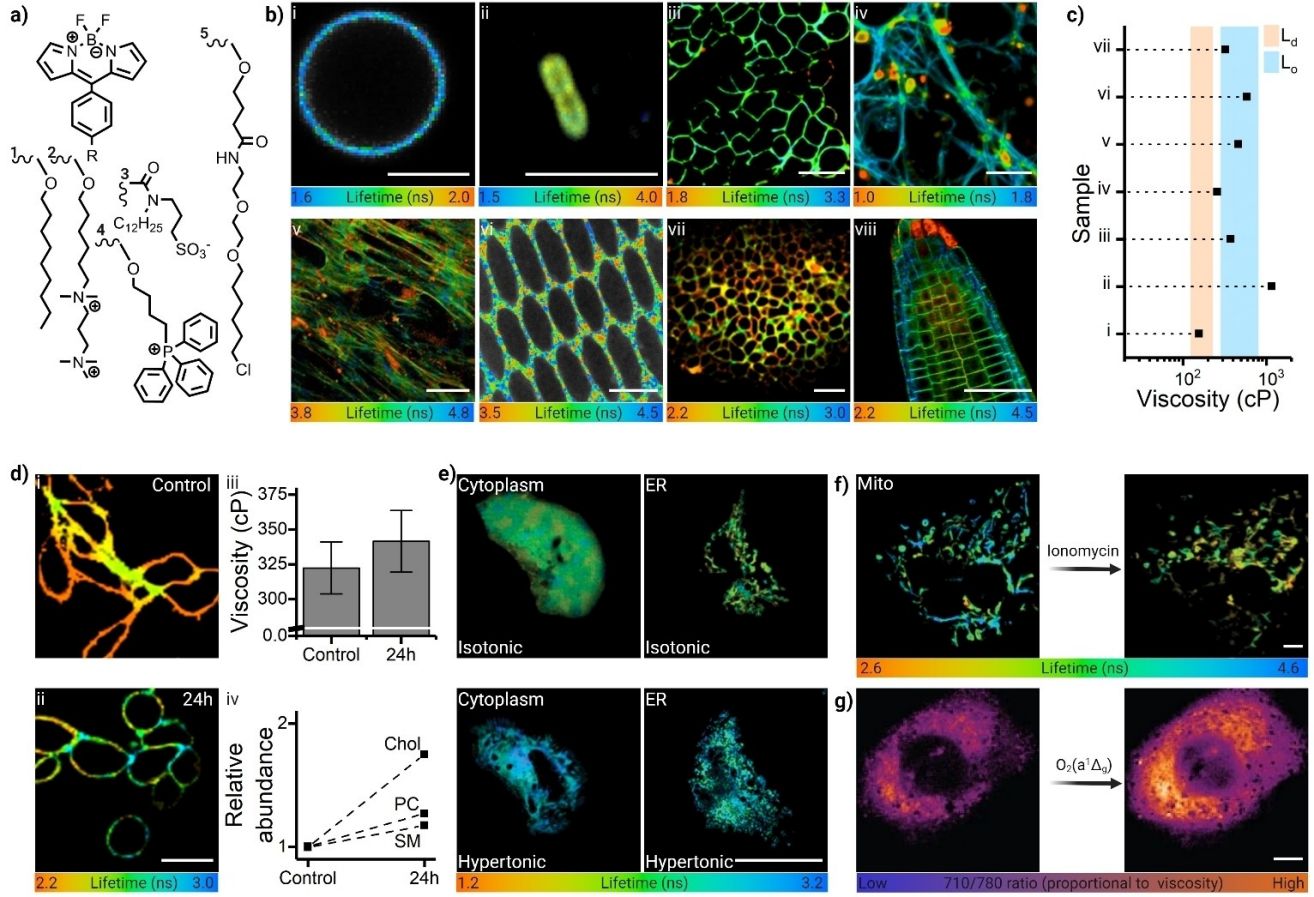
Targeted molecular rotors report on the local microviscosity in organelles of live cells. a) Example targeting moieties used to stain the cell organelles: (1) untargeted;^[48a]^ (2,3) plasma membrane;[^50^, ^108^] (4) mitochondria;^[109]^ (5) Halo‐tag.^[31]^ b) FLIM images illustrating the use of BODIPY‐based molecular rotors to quantify the microviscosity of lipid membranes in (i) Giant plasma membrane derived vesicles (GPMVs) (Adapted with permission from ref. [96] Copyright 2019, Springer Nature) (ii) inner membrane of E. Coli, (Adapted with permission from ref. [60] Copyright 2016, Elsevier) (iii) SH‐SY5Y cells (Adapted with permission from ref. [68] Copyright 2018, Royal Society of Chemistry) (iv) 2 DIV neurites (Adapted with permission from ref. [67] Copyright 2019, American Chemical Society) (v) chondrogenic differentiating mesenchymal stem cells (Adapted with permission from ref. [58b] Copyright 2020, Springer Nature) (vi) porcine eye lens (Adapted with permission from ref. [104] Copyright 2021, Elsevier) (vii) HeLa tumour spheroids (Adapted with permission from ref. [103] Copyright 2020, Society of Photo‐Optical Instrumentation Engineers—SPIE) and (viii) Arabidopsis roots plasma membranes (Adapted with permission from ref. [58a] Copyright 2019, National Academy of Science) Images were adapted from the corresponding refs. Scalebar: 5 μm (i,ii,vi), 50 μm (iii,iv,v,vii,viii). c) FLIM‐derived viscosity values for the images shown in (b). Shaded areas represent the range of viscosity values typical of L_d_ and L_o_ domains in synthetic lipid membranes. d) BODIPY molecular rotor measures increased viscosity in cisplatin‐treated HeLa cells at 24 h (i shows control and ii 24 h treated HeLa cells with 2.6 μM cisplatin). Scalebar: 50 μm. Calculated viscosity changes (iii) can be linked to alterations in the lipid composition, quantified via mass‐spectrometry imaging (iv). (Adapted with permission from ref. [103] Copyright 2020, Society of Photo‐Optical Instrumentation Engineers—SPIE) e,f) Halo‐Tag technology allows selective targeting of BODIPY‐based molecular rotors towards distinct cellular organelles, enabling an accurate determination of organelle's viscosity and responses under stress; e.g.: cytoplasm and ER show increased viscosity under hypertonic conditions (increased osmotic stress). Scalebar: 50 μm. (Adapted with permission from ref. [31] Copyright 2018, American Chemical Society) f) Mitochondrial Halo‐Tag labelling shows increased mitochondrial viscosity upon ionomycin treatment. Scalebar: 5 μm. (Adapted with permission from ref. [31] Copyright 2018, American Chemical Society) g) Spectral ratiometric imaging using porphyrin dimer‐based rotors reveals a large increase in organelle viscosity during photodynamic therapy (PDT). Scalebar: 5 μm. (Adapted with permission from ref. [45a] Copyright 2009, Springer Nature).

When selecting an appropriate molecular rotor it is important to consider its hydrophobilicy/hydrophilicity, propensity to self‐aggregate or bind biomolecules, relative brightness and the nature of its excited state decay (i.e. single component decays require less photons to be collected and are easier to interpret). Meso‐phenyl substituted Boron‐dipyrrin (BODIPY) lifetime‐based molecular rotors (Figures 1e and 3a) received significant attention due to their favourable photophysical properties, such as high quantum yield, narrow emission profiles, and monoexponential time‐resolved decays that simplify fitting. Most importantly, the response of BODIPY probes to other confounding parameters, such as polarity and temperature is well characterised,^[29a]^ also see *Temperature measurement* section. These dyes were well suited for measurements of the microviscosity of hydrophobic environments, such as lipid membranes in model[^55b^, ^57^] and cellular[^39^, ^58^] systems, cellular organelles in mammalian[^31^, ^59^] and bacterial cells,^[60]^ in atmospheric aerosols,^[61]^ self‐assembled peptides,^[62]^ and when exploring polymerisation mechanisms,[^8b^, ^63^] to mention a few (Figures 2–3 and 5). While some of these dyes exhibit fast cellular internalisation, synthetic strategies can be employed to increase a dye's residence time in the membrane, e.g. by incorporating an amphiphilic anchor with significant positive charge, Figure 3a.^[64]^ BODIPY‐based rotor dyes were used to investigate the plasma membrane microviscosity of differentiating stem cells,^[58b]^ of cancer cells (both in vitro^[65]^ and in vivo^[66]^), of neuronal membranes subjected to neurodegenerative stimuli,^[67]^ and of plasma membranes affected by the presence amyloid beta (Aβ) aggregates.^[68]^ Targeted internal cellular organelle staining can also be achieved. Cationic triphenyl‐phosphine anchors facilitate BODIPY accumulation in the mitochondria,^[69]^ and ethynylestradiol or morpholine groups can be used to target the endoplasmic reticulum.[^59^, ^69c^, ^70^] More recently, *Ashokkumar* et al. combined a BODIPY rotor and a zinc‐dipicolylamine to create a biosensor for apoptosis,^[71]^ and *Michels* et al. also demonstrated that BODIPY rotors modified with solubilising or targeting groups could be used to investigate the microviscosity of plant tissues and their mechano‐chemical response.[^58a^, ^72^]

On the other hand, several families of hydrophilic molecular rotor dyes, with lifetime‐based responses to viscosity, were successfully used to probe the crowding in the predominantly aqueous environment of cellular organelles,[^31^, ^73^] in semi‐solid secondary organic aerosols,^[61b]^ and to probe the mechanisms of amyloid aggregation,^[74]^ amongst others.^[62a]^ The Cy3‐based structures (initially proposed by Luby Phelps for measuring cytoplasmic viscosity by ratiometric method, in combination with viscosity‐insensitive Cy5) are extremelly useful as hydrophilic FLIM‐based molecular rotors,^[41]^ although their polarity or temperature sensitivity has not been investigated. Thioflavin T, a widely used probe for amyloid fibers,^[75]^ was demonstrated to work as a molecular rotor in solutions of increasing viscosity,^[76]^ and was shown to sense microscopic confinement and crowding during amyloid protein aggregation, as long as it was not strongly bound to amyloid beta‐sheets.[^74a^, ^77^] As such, it was well suited for probing the aggregation mechanism of insulin, but not that of lysozyme.[^74a^, ^77^]

Finally, recent progress in genetic targeting motifs allowed Halo[^31^, ^78^]‐ and SNAP‐tagged[^73^, ^79^] rotors to be directed to selected cellular organelles (Figure 3e,f), and to study disease‐related biological crowding responses, e.g. abnormalities associated with z‐antitripsin misfolding in the endoplasmatic reticulum, ER.^[62c]^

Given a large breadth of potential application of molecular rotors, we do not strive to cover this field in full. Instead, we will focus on highlighting molecular rotor capabilities under developement in our group, and will draw comparisons to the current state of the art methods for imaging viscosity, diffusion and macromolecular conformation.

## 3 Molecular rotors for biological imaging

### 3.1 Measuring membrane viscosity using molecular rotors

Since viscosity is one of the major determinants of molecular diffusion, it is thought to be of particular importance in lipid membrane function, both in the normal cell function and disease, and in drug delivery. However, it is challenging to probe membrane viscosity non‐invasively, and with required spatial resolution, to reflect the potential heterogeneity present. While a myriad of different dyes have been developed to study the microenvironment of lipid membranes,^[80]^ they often respond to multiple biophysical features and/or are not fully calibrated against the various membrane's parameters.

One of the key advantage of using molecular rotors is the possibility of obtaining quantitative, dynamic maps of the membrane viscosity. This is crucial when studying heterogeneous lipid membranes showing phase separation, Figure 2a,b. Typically, membranes containing a mixture of saturated and unsaturated lipids, and cholesterol, will phase separate into disordered domains (rich in unsaturated lipids) and more ordered regions (composed of saturated lipids and cholesterol); minimizing the line tension arising from the hydrophobic mismatch between the two lipid types. The later, more ordered domains, are representative of “lipid rafts” which are believed to play a central role in signal transduction and protein organization and are, therefore, of high biological interest.^[81]^ The use of molecular rotors allowed the detection of liquid ordered (L_o_, lipid raft‐like) and liquid disordered (L_d_)—like phases, in plasma membranes of live cells and in model membranes (Figure 2b), under conditions of oxidative attack[^57c^, ^83^] (Figure 2e) and osmotic stress^[55b]^ (Figure 2f). While in model systems the L_o_ and L_d_ domains can be clearly distinguished by diffraction‐limited micoscopy, the lipid raft domains are often too small to detect directly in live cells, yet their formation can be inferred from the colocalisation of lifetime signals of distinct phases (see Figure 3b,c), or via the phasor‐analysis.[^57b^, ^84^]

Apart from the variations in lipid composition, e.g. leading to the formation of L_o_ and L_d_ phases, external interactions of lipid layers either between themselves or with exogenously added (macro)molecules were shown to alter their viscosity. We demonstrated that touching lipid‐coated microbubbles show a significantly higher viscosity at the point of contact (Figure 2c), presumably, due to screening of monolayer interactions withwater, while an addition of a hydrophilic PEG polymer coating to the lipid reduces the observed viscosity of the bubble shell.^[22]^ Similarly, touching live cells grown as a monolayer exibited higher membrabe viscosity at the point of contact, compared to “free” membranes.^[85]^ We also demonstrated that the addition of aggregated amyloid beta (Aβ) oligomers reduces the bilayer viscosity in GUVs (Figure 2d) and in live SH‐SY5Y cells, while the addition of fibrillar aggregates of Aβ increases the bilayer viscosity.^[68]^ While smaller oligomeric species are likely to be able to insert into the bilayer, at least to some extent, larger fibrillar species are expected to localise externally, and to increase the membrane order indirectly, e.g. by their interactions with lipid heads.

It has been verified that the viscosity values that were derived from molecular rotor lifetimes in lipid membrane systems correlate well with the packing of a lipid phase (e.g. $\tau_{gel}>\tau_{L_{o}}>\tau_{L_{d}}$ ) and with the diffusion coefficients, measured directly by FCS and calculated via molecular dynamics (MD) simulations.[^57a^, ^57c^] We were able to demonstrate recently that BODIPY‐molecular rotor‐derived viscosities correlate strongly with structural membrane parameters determined by X‐ray crystallography (Figure 2g). Small Angle X‐Ray Scattering (SAXS) provides information about interlamellar spacing, tilt angle, or membrane thickness (d_HH_); while Wide Angle X‐Ray Scattering probes the in‐plane membrane organisation, providing information about the lipid phase or the area per lipid (APL).^[86]^ These analytical correlations between d_HH_/APL and viscosities measured by rotors open possibilities to obtain structural parameters from membranes of live cell, which are traditionally only available from SAXS and WAXS measurements in bulk samples.^[82]^

Molecular rotors are commonly compared to Laurdan, which is, perhaps, the most commonly used commercial solvatochromic probe to report on membrane's properties and lateral organisation.^[87]^ The dipole moment along Laurdan's naphthalene moiety changes depending on the polarity of the local environment, thus responding to a more hydrated environments by fluorescence spectral shift towards longer wavelengths^[88]^ (the so call “Laurdan General Polarization function”, GP)^[89]^ and shorter fluorescence lifetimes.[^87b^, ^90^] While hyperspectral measurements can be quicker than lifetime acquisition, the multiparametric dependency of Laurdan's fluorescence makes it a non‐ideal candidate to unequivocally probe the viscosity and packing of lipid bilayers. While in molecular rotor‐based approaches there is only one physical parameter being probed—viscosity. This single membrane's molecular architecture parameter dependency is an advantage, compared to Laurdan‐based approaches.

Second‐generation GP‐based probes include Nile Red derivative NR12S,^[91]^ the styryl‐based dye di‐4‐ANEPPDHQ,^[92]^ or 3‐hydrocychromone based dyes such as F2N12S or F66NS.[^18a^, ^93^] The use of these probes enabled the monitoring of membrane dipole potential (di‐4‐ANEPPDHQ),^[94]^ or simulataneously measuring polarity and hydration of lipid bilayers,^[95]^ which are linked to bilayer packing and viscosity.^[96]^

On the other hand, *Flipper* dyes (e.g. commercially available FlipTR® ) developed by *Matile* and colleagues are extremelly popular probes of lipid order via FLIM.^[80c]^ This family of push‐pull probes has been developed as sensors of membrane tension and enabled the evaluation of its effects in a myriad of cellular processes, such as protein‐membrane interactions,^[97]^ organelle‐specific response to tension,^[98]^ endocytosis^[99]^ or mitochondrial fission.^[100]^ While FlipTR® are assumed to depend on tension alone, their responses appear to be lipid‐composition dependent and, as such, require individual calibrations tailored to each membrane in order to extract physically meaningful quantities of membrane tension.^[18a]^ On the other hand, BODIPY rotors, whose lifetime‐viscosity relationship can be extracted from a single calibration curve, report on viscosity/packing, which automatically takes membrane composition into account.[^57a^, ^101^]

The combined use of molecular rotor‐based FLIM and MD simulations in lipid membranes[^57a^, ^57c^] gave us confidence in the fact that the rotors do not relocalise, i.e. do not change height of tilt angle, in membranes of different composition (but the same phase)—and, thus, their lifetime signal responds to lipid chain packing, rather than an altered position within the lipid chains. Further, MD simulations allowed us to calculate expected diffusion coefficients in membranes of different composition and at a different temperature.[^57a^, ^57c^] Thus, for example, we were able to unravel the role of lipid peroxidation on the membrane's viscosity (Figure 2e). This process plays a key role in cell signalling and disease, and it is involved in photodynamic therapy (PDT, a form of cancer treatment). Traditionally it has been accepted that the presence of a hydrophilic ‐OOH group will disrupt the membrane's structure and lower its rigidity.^[102]^ However, a combination of molecular rotors and FLIM microscopy revealed that peroxidation was not accompanied by a concommitant decrease in membrane viscosity.^[83]^ Instead, we observed an uncoupling of the membrane's viscoelastic properties, i.e. a previously documented decrease in bending rigidity^[102]^ and a new observation of an increase in viscosity, upon peroxidation, which was caused by emerging H‐bond interactions between the peroxidized lipids.^[57c]^ Using FLIM we were also able to detect the formation of microscopic lipid clusters of increased viscosity (similar to “lipid rafts”) in single‐component oxidised membranes, which were also detected in MD simulations, Figure 2e.^[57c]^

**Figure 4:**
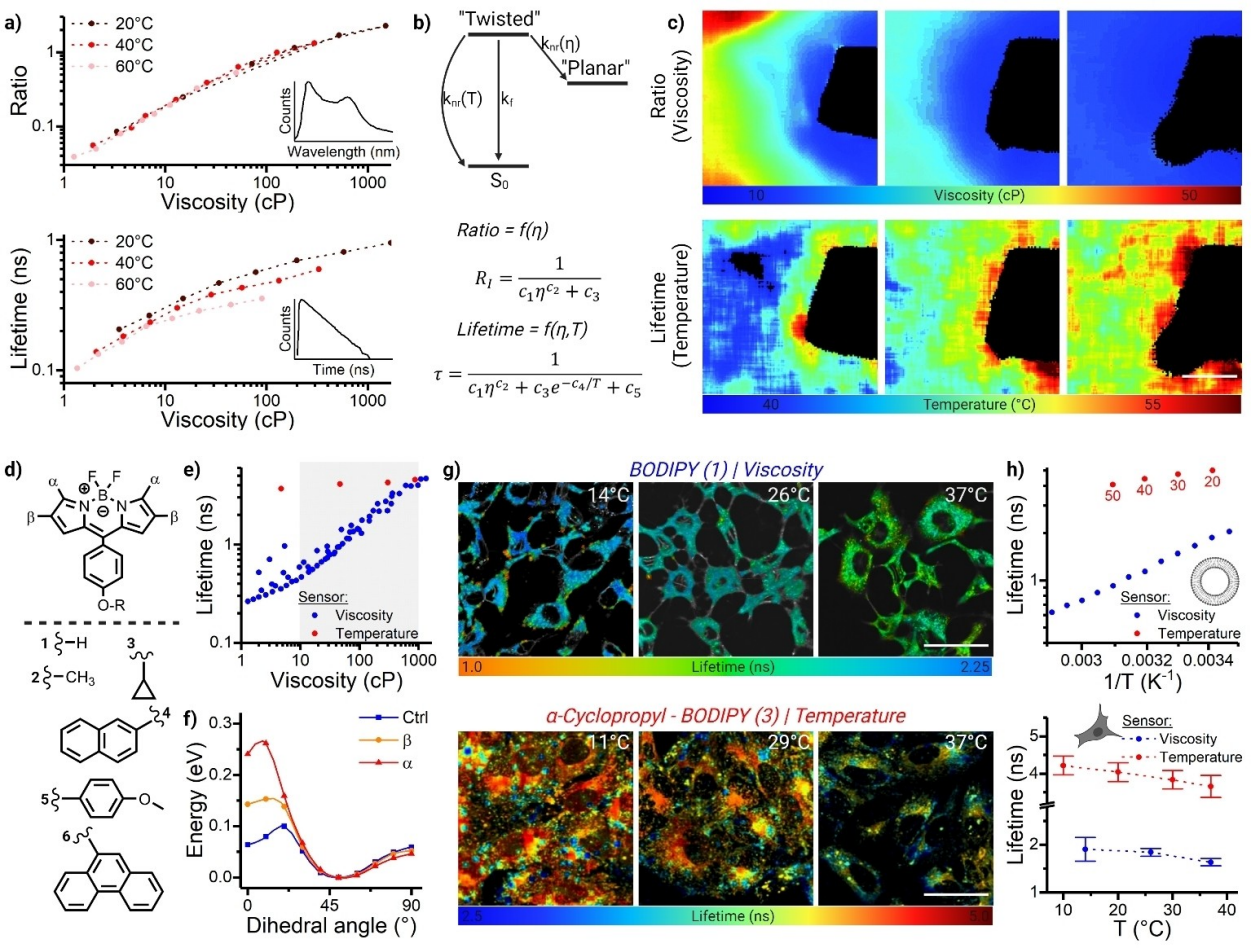
Temperature imaging using molecular rotors. a) Spectral ratiometric (top) and lifetime (bottom) calibration of porphyrin dimer‐based rotor (Figure 1d) at different temperatures and viscosities in methanol/glycerol mixtures. While the ratiometric readout shows excellent overlap of all points and, hence, no sensitivity to temperature, the lifetime data is temperature‐dependent since the data points recorded at different temperature do not overlap. b) Dual viscosity/temperature sensitivity of the dimer arises from the temperature‐dependent non‐radiative deactivation of the twisted excited state, k_nr_(T), which competes with viscosity‐dependent conversion between the *twisted* and the *planar* conformations following excitation, k_nr_(η). Viscosity sensitivity of the ratiometric data can be well described by a Hill's function, and the lifetime sensitivity to both parameters can be described by the addition of an Arrhenius term.^[55a]^ c) Combined ratiometric and FLIM imaging of porphyrin dimer‐based probes allows simultaneous imaging of viscosity and temperature gradient induced by a heated copper wire, using equations shown in b). Scalebar: 50 μm. (Adapted with permission from ref. [46b] Copyright 2015, Royal Society of Chemistry) d) Extending the conjugation in BODIPY‐based viscosity sensors using the substituents shown below results in temperature sensitivity. e) Lifetime‐based calibration of a classic non‐substituted rotor (Phenyl‐BODIPY, blue), as compared to α‐cyclopropyl BODIPY (red), the latter showing no sensitivity to viscosity. (Adapted with permission from ref. [51] Copyright 2021, American Chemical Society) f) Calculated potential energy surfaces of the S_1_ state of cyclopropyl probes shown in d). A higher energy barrier for *m*‐phenyl group rotation results in lower viscosity sensitivity, converting molecular rotor dye into a temperature probe instead. Note, the lifetimes of β‐cyclopropyl BODIPY (not shown) display dual sensitivity to viscosity and temperature owing to an intermediate height of the energy barrier, and this dye cannot be used to probe either parameter. (Adapted with permission from ref. [51] Copyright 2021, American Chemical Society) g) FLIM images of U2OS cells stained with a viscosity (Phenyl‐BODIPY, top) or temperature (cyclopropyl, bottom) shown in d), at different temperatures. Scalebar: 50 μm (Adapted with permission from ref. [51] Copyright 2021, American Chemical Society) h) Comparison in the measured parameters between viscosity‐sensitive (Phenyl‐BODIPY) and α‐cyclopropyl substituted BODIPY probes in model DOPC (top) and cellular (bottom) membranes. Both parameters can be measured with acceptable sensitivity, although the dynamic range of viscosity measurements is higher than that of temperature. (Adapted with permission from ref. [51] Copyright 2021, American Chemical Society and ref. [101] Copyright 2013 Royal Society of Chemistry).

One unique property of using BODIPY‐based molecular rotors for determination of lipid membrane behaviour is their sole sensitivity to viscosity, rather than the temperature, polarity, or hydration^[29a]^ of these membranes. This property made it possible to use molecular rotors to directly visualise and compare plasma membrane viscosities of different cell types (Figure 3b,c),[^31^, ^48a^, ^50^, ^65^, ^84^, ^103^] at different conditions.[^39^, ^103^, ^104^] For example, we were able to follow the viscosity changes of U2OS cells with temperature (Figure 4f), and establish that the plasma membrane of primary neurons exibit the lowest levels of viscosity detected to date (Figure 3b‐iv).[^67^, ^68^] At the same time, the inner membrane of bacterial spores was characterised by extremelly high viscosity^[60]^ (equivalent to a gel phase in model lipid bilayers), whereas plasma membranes of porcine eye lens cells appeared to be purely in the L_o_ raft‐like phase.^[84]^ These viscosities can be further altered by treatment, e.g., chemotherapy of cancer cells, Figure 3d,[^58c^, ^103^, ^105^] photooxidation, Figure 3f,[^45a^, ^104^] (e.g. during PDT) or alcohol‐ and temperature‐based deactivation of bacteria.^[106]^ In some cases it was possible to directly correlate the viscosity changes to specific lipid composition in the plasma membranes, detected by mass‐spectroscopy imaging[^58b^, ^58c^] (Figure 3d) or by preparing synthetic oxidised membranes of known composition.^[57c]^ While in other cases the viscosity change was brought about by external interactions, e.g. absorption of polymers on lipid‐based ultrasonic microbubbles,^[22]^ or plasma membrane interaction with toxic oligomers and fibrils of amyloid‐beta, Figure 2d.^[68]^

However, despite the advantages afforded by BODIPY‐based rotors, their preferential partitioning towards disordered lipid phases[^57a^, ^101^] remains a challenge when investigating cellular membranes. The nanometre‐lengthscale highly‐ordered lipid clusters present in these structures are below the diffraction limit, and therefore cannot be optically resolved. Thiophene‐based molecular rotors developed by *Lopez‐Duarte* et al.^[107]^ have equal partitioning regardless of the lipid phase, and therefore their readout is less influenced by the membrane's heterogeneity.^[57b]^ We used these molecular rotors to demonstrate the coexistence of more ordered raft‐like domains with more fluid membrane regions in live cultured cells of different types,^[57b]^ and to investigate the mechanical behaviour of model lipid membranes subjected to tensile or compressive forces, Figure 2f.^[55b]^ Our results, alongside others,^[18a]^ provided experimental evidence of several types of non‐canonical responses to tension in lipid bilayers, driven by the presence of highly‐ordered lipid domains, including tension buffering, strain‐hardening or pressure‐induced softening; and these behaviours could play a role in malaria infection^[110]^ or in the auditive process.^[111]^

Precise organelle microviscosity imaging utilised an irreversible covalent reaction of Halo‐[^31^, ^62c^, ^78b^] or SNP‐^[73]^ tags conjugated to molecular rotors, with genetically encoded proteins expressed in a specific live cell compartment. This approach allowed to compare viscosities/crowding of individual cellular organelles, as well as to detect the effect of various types of perturbation on the cellular viscosity hoteostasis, such as osmotic stress (Figure 3e), drug treatment^[31]^ (Figure 3f) and abnormal health conditions, e.g. overexpression of z‐antitripsin in the ER during the unfolded protein response.^[62c]^ Genetic targeting approaches have the advantage of knowing the precise localisation of the rotor in the organelles of interest, although good results can be also achieved with non‐covalent targeting ligands, Figure 3a, such as triphenylphosphine (for mitochondria),[^69b^, ^109^] or morpholine (for lysosomes).^[70]^ Although not covered here in detail, other rotor dyes have also been used to investigate the microviscosity of lipid membranes and intracellular compartments; through intensity, ratiometric, or lifetime‐based measurements. These include merocyanine,^[112]^ anthracene,^[113]^ triphenylamine,^[114]^ juliodine,[^32^, ^33^] porphyrin,[^45a^, ^46b^] pyrrolic compounds,^[115]^ GFP,^[87a]^ or indocarbocyanine nanoparticle^[116]^—based molecular rotors.

### 3.2 Measuring temperature using molecular rotors

As with many other environmentally sensitive dyes, temperature has the potential to play a large role in the measured signal of molecular rotors. Firstly, temperature can alter the viscosity of the environment, with particularly large effects seen in the viscous environment. E.g. by changing the temperature between 15 and 37 °C, the viscosity of water only changes between 1.14 and 0.70 cP, however, the viscosity of glycerol changes between ca. 2280 and 350 cP.^[117]^ 1,2‐Dioleoyl‐sn‐glycero‐3‐phosphocholine (DOPC) bilayers change viscosity between ≈250 and 60 cP^[101]^ as measured by variations in BODIPY rotor's lifetimes. But how can we ensure that the changes in lifetime, seen from the rotors in viscous membranes and in cells, are due to true changes in the bilayer's viscosity, and not to the effect of temperature on the photophysics of the rotor itself?

By recording rotor's lifetimes or ratiometric signal in solutions of different composition at different temprature it is possible to directly verify the role of temperature. In homogeneous methanol/glycerol solutions the bulk viscosity can be measured for every temperature of interest using bulk rheometry. Then the lifetime or ratios of the rotors can be plotted against this known viscosity,[^29a^, ^55a^, ^101^] Figure 4a. The lifetimes recorded at different compositions and temperatures for BODIPY rotors overlap perfectly, indicating that this sensor's lifetime responds to the viscosity alone and thus can be used to measure viscosity, e.g. in the lipid bilayers, at varied temperature, Figure 4e. The spectral ratios recorded for the series of porphyrin dimers also show temperature and composition‐independent responses, Figure 4a.^[55a]^ However, many other rotor fluorophores, such as Kiton Red^[55a]^ (Sulforhodamine B) and thiophene‐based molecular rotors^[55b]^ show viscosity‐ and temperature‐dependent lifetimes, where the effect of both parameters can not be easily decoupled. One solution is to calibrate the probe and measure the viscosity at a fixed temperature only (e.g. 37 °C, needed for live cell work). It is important to note that the reverse measurement (e.g. measuring temperature at a fixed viscosity) is not feasible, because variations in temperature dramatically alter the viscosity, especially in crowded environments, which are often found in biological systems.

While the ratiometric signal of all the porphyrin dimers studied was insensitive to the temperature of their environment and responded to viscosity alone, the lifetime of some of the dimers, recorded in the “twisted” conformer peak, showed a puzzling dependence on both the temperature and viscosity,^[55a]^ Figure 4a. By fitting this data to the Hill's function with an additional Arrhenius parameters, Figure 4b, we were able to establish the activation energy associated with the un‐twisting and/or the non‐radiative decay of the dimer to the ground state. Thus, even though the lifetime decay of the dimers depended on both the temperature and viscosity, the temperature dependency can be very well understood and predicted, provided the viscosity is known. Since both signals—ratiometric and lifetime—can be measured simultaneously, this allowed the parallel imaging of both properties, with good precision, using two‐channel spectral detection and FLIM in a standard confocal microscope, Figure 4c.[^21^, ^55a^]

We also discovered that the temperature‐insensitivity is not a universal property of all BODIPY rotors. We synthesised series of BODIPY rotors bearing cyclopropyl,^[51]^ phenanthrene or naphthalene^[118]^ substituents (Figure 4d), with the aim of increasing the delocalisation through the BODIPY core and achieving red‐shifted emission of the rotor. However, these substitutions have resulted in increasing of the energy barrier between the bright (emissive) and the dark states of the rotors, Figure 4f. At low values of activation energy (0.10 eV, for parent green‐emitting BODIPY rotor) the temperature was found not to play a role, Figure 4e; however, for intermediate activation energy values (0.15 eV, e.g. for β‐cyclopropyl substituted rotor) both viscosity and temperature were shown to affect the observed lifetimes.^[51]^ Interestingly, for the high activation energy case (0.26 eV, for e.g. for α‐cyclopropyl substituted rotor) viscosity was found not to play a role, and instead the decay of this rotor in low‐polarity environment was completely determined by temperature (Figure 4e, red data points). Based on these principles, our group^[51]^ and others^[119]^ utilised BODIPY‐based “molecular thermometers” for measuring temperature in hydrophobic and non‐polar organelles of live cells, at varied temperature, Figure 4g,h.

It is worth noting that other modifications to the periphery of BODIPY chromophores that do not directly affect the rotation of the meso‐phenyl group were reported to alter the rotor's photophysics. Methyl substituents in the alpha and beta positions of the BODIPY were shown to reduce the dynamic range of the rotor's responses to viscosity, and were assigned to the formation of the butterfly conformations of the dye.^[120]^ Extending conjugation of the BODIPYs in the alpha‐ and beta‐ positions by introducing double and triple bonds were shown to completely remove the sensitivity of the dyes to temperature and viscosity, in limiting cases resulting in dyes with fluorescence quantum yields close to unity, which is a very sought‐after property in a red‐shifted BODIPY analogues, albeit not environmentally sensitive.[^29a^, ^121^] On the other hand, it has been shown that the addition of electron‐withdrawing groups, such as ‐NO_2_ ^[122]^ or ester^[29a]^ in the para‐position of the phenyl ring of the BODIPY can expand its dynamic range of viscosity sensitivity over two orders of magnitude without leading to an undesirable additional temperature sensitivity.

### 3.3 Measuring protein aggregation using molecular rotors

**Figure 5:**
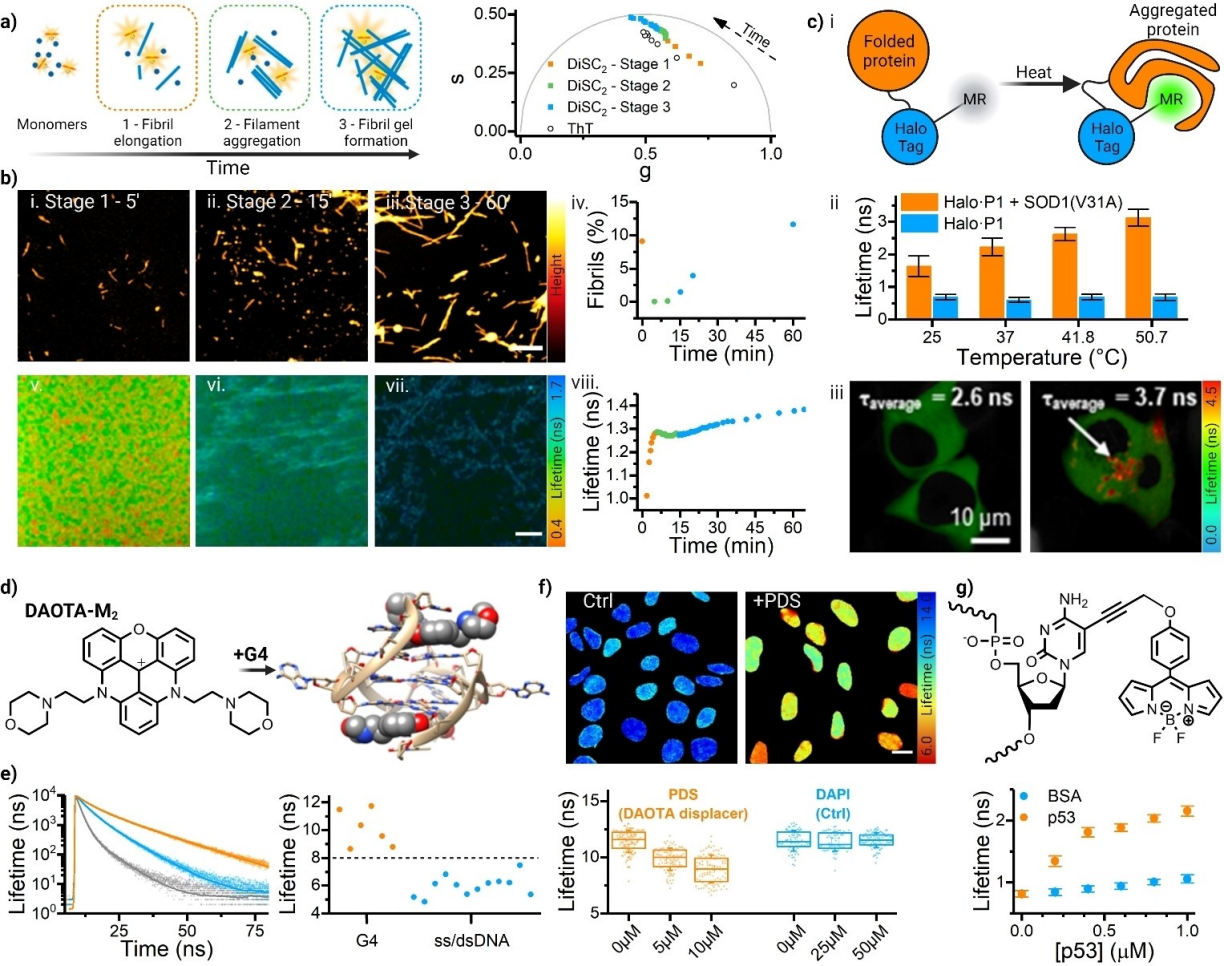
Molecular rotors measure protein aggregation and nucleic acid conformation. a) Lysozyme aggregation is a multistep process, which can be tracked via the phasor analysis of time‐resolved decays of molecular rotors 3,3‐Diethylthiadicarbocyanine (DiSC_2_, filled squares) and Thioflavin T (ThT, hollow circles). While ThT reports on the fibril formation, it displays limited sensitivity to other intermediates, as shown by a linear phasor trajectory showing conversion between monomeric/fibrillar lysozyme. DiSC_2_, on the other hand, is sensitive to more than two states in the mixture, as evidenced by a non‐linear phasor trajectory. (Adapted with permission from ref. [74a] Copyright 2015, American Chemical Society) b) AFM images of aggregating lysozyme mixture with aliquots taken at 5 min (i), 15 min (ii) and 60 min (iii) since the seed addition and nanopore current recordings (iv) showing % fibril present. This data correlate with FLIM measurements of the same mixture upon the addition of molecular rotor DiSC_2_ (v–vii). The lifetime evolution of DiSC_2_ (viii) shows a matching trajectory to nanopore current readings shown in (iv) and the low lifetime recorded at Stage 2 of aggregation. Note, lower lifetime shown by green symbols in viii and low lifetime seen in FLIM in panel (vi) coincide with appearance of small circular aggregates seen in AFM (panel ii). Scalebar: 500 nm for (i–iii) and 100 μm for (v–vii). (Adapted with permission from ref. [62b] Copyright 2019, American Chemical Society and ref. [74a] Copyright 2015, American Chemical Society) c) Fluorescence lifetime from Halo‐Tag protein‐bound molecular rotors can be used to investigate protein aggregation *in cellulo*: cartoon representation (i); increased lifetime of molecular rotor is detected in P1 in the presence of SOD1(V31A), due to heat‐induced protein aggregation, but not for P1 control in vitro (ii) and *in cellulo* (iii). (Adapted with permission from ref. [129] Copyright 2022, Wiley‐VCH GmbH) d) The DAOTA‐M_2_ is a viscosity‐sensitive rotor that binds strongly to G‐quadruplex (G4) DNA. e) DAOTA‐M_2_ lifetime is significantly higher upon G4 binding compared to duplex (ds) and single stranded (ss) DNA. (Adapted with permission from ref. [130] Copyright 2021, Springer Nature) f) FLIM imaging of G4 binding of DAOTA‐M_2_ in live cells: (i) FLIM images recorded for control cells and those incubated with a strong G4 binder pyridostatin (+PDS). Scalebar: 20 μm. (ii) Lifetime analysis of control cells and those incubated with increasing concentrations of PDS (reduction in lifetime is observed consistent with DAOTA‐M_2_ displacement from G4s by PDS) and with DAPI, a nuclear stain that binds to duplex DNA and does not displace DAOTA‐M_2_. (Adapted with permission from ref. [130] Copyright 2021, Springer Nature) g) A BODIPY‐based rotor covalently linked to DNA enables the specific recognition of DNA binding to p53 protein. No change is observed in the presence of BSA (non‐specific binder). (Adapted with permission from ref. [131] Copyright 2015, Wiley‐VCH GmbH).

As we have seen above, molecular rotors are sensitive to the free solvent volume in their immediate environment. This property is ideal for microviscosity measurements in samples such as hydrophobic tail region of the lipid bilayers,[^55b^, ^57a^, ^82^, ^101^] in ionic liquids,^[123]^ or in atmospheric aerosol samples.^[61]^ However, the effect of microconfinement on the rotor's lifetimes and ratios can also be exploited to investigate macromolecular crowding that occurs during polymerisation in synthetic[^7^, ^8b^, ^63^, ^124^] and natural polymers,^[125]^ as well as ligand‐protein binding^[126]^ and protein aggregation, folding, and interaction,^[127]^ where the concept of viscosity is less well defined.^[128]^ However, due to the possibility of molecular rotor binding to the target protein, care should be taken when interpreting such data, especially when the measurement is intensity‐based. Upon binding, the conformational freedom of a molecular rotor will be restricted and this will result in higher fluorescence signal and/or lifetime. Lifetime‐based measurements usually allow to distinguish between binding events and a true increase in crowding. For example, ThT, a widely used amyloid stain,^[75]^ can act as a molecular rotor, displaying increased lifetime in higher glycerol/water mixtures.^[76]^ ThT was shown by lifetime‐based approaches to report on crowding during insulin aggregation. However, due to the strong specific binding to lysozyme fibrillar structures, ThT was only able to report on the presence of fibrillar aggregates during lysozyme aggregation, as shown by a linear phasor trajectory during aggregation, Figure 5a. This linear trend indicated that ThT detects interconversion of two ThT states only: i.e. ThT free in solution (short lifetime) and ThT bound to fibrils of lysozyme (long lifetime), (Figure 5a).[^74a^, ^77^]

In contrast, in the absence of strong binding between the protein and the rotr, FLIM measuremets can reveal details of the mechanism of amyloid aggregation. E.g. molecular rotor DiSC_2_ was able to report on the details of dynamics of the supremolecular assembly of amyloid proteins, such as Aβ, insulin[^62a^, ^77^] and lysozyme.[^62a^, ^74a^] This was possible due to the sensitivity of this rotor to the restrictions of the solvent‐free volume. We were able to demonstrate that the seeded aggregation of lysozyme proceeds via fragmentation process, Figure 5a,b, leading to reduced viscosity seen at early aggregation stages, at short time scales that were not considered feasible for fragmentation previously. The trend can be clearly seen in the phasor representation, by a non‐linear aggregation trajectory, Figure 5a, confirming that the rotor is sensitive to the presence of additional aggregating species, not just the fibrils. A reduction in lifetime (indicative of a reduction in crowding) is clearly seen in DiSC_2_ rotor lifetime recorded as a function of time since seeding the aggregation mixture (Figure 5b‐viii). This lifetime dip coincides with the current blockage trend recorded in nanopore electrical measurements of the same aggregation mixture (Figure 5b‐iv), interpreted as % fibrillar aggregates present. We additionally confirmed the presence of small fragmented oligomer‐like species in the same lysozyme aggregation mixture by AFM (Figure 5b‐i–iii) seen on the same timescales as the reduction in crowding sensed by molecular rotor measurements and nanopore detection.^[62b]^

Molecular rotors have been used to investigate the formation of amyloid beta (Aβ) aggregates, which play a major role in neurodegenerative pathologies such as Alzheimer's disease.^[132]^ The use of BODIPY,[^62a^, ^133^] cyanine[^62a^, ^74a^] and ThT[^74a^, ^75^, ^134^]—based rotors allowed the in vitro and *in cellulo* monitoring of the Aβ aggregation process. Importantly, covalent attachment of rotors to an aggregating monomer of interest was necessary to enable the monitoring in live cells, to ensure that the rotor continues to co‐localise with the protein even after the incubation *in cellulo*. Interestingly, we were able to demonstrate that the mechanism of Aβ aggregation differs significantly in vitro compared to that in the presence of live cells. In the latter case, we believe the interaction of Aβ with cellular membranes causes a large initial reduction in protein crowding.^[62a]^ In a separate experiment, we were able to verify that the presence of Aβ aggregation products also affects cellular and model membranes, Figure 2d. Oligomers caused a reduction in the lipid bilayer's viscosity, whereas Aβ fibrils’ assocation with cellular and synthetic membranes resulted in an increased viscosity values of these bilayers.^[68]^

Molecular rotors have also been employed to measure the aggregation dynamics of other proteins such as collagen,^[125b]^ antibodies,^[135]^ or dismutases (Figure 5c).^[129]^ In the latter case, Halo‐targeting allows to colocalise molecular rotor with the protein of interest and perform the measurements of aggregation in live cells. Recently, *Liu* and colleages developed red‐fluorescent protein based,^[136]^ non‐covalent, cell‐permeable proteome aggregation sensor capable of reporting the assembly and clearance of protein aggregates.^[137]^ The team later expanded this technology for the multiplexed detection of protein aggregates, providing insight into the influence of protein co‐aggregation *in cellulo*.^[138]^

Altogether, these examples highlight the capability of molecular rotors to probe mechanisms of protein aggregation, (mis)folding and crowding. However, unlike in lipid membranes, the crowding effects of protein microenvironment are far less understood, and, therefore, stringent controls are necessary to draw a complete picture of how the rotors interact with biomolecules, e.g. through course‐grain/atomistic simulations. Such work can reveal not only the mechanism of protein‐rotor conjugate's action, but also help unravel the possible effects of the protein environment on the rotor. We were able to observe a pronouced photo‐bleaching effect in Halo‐BODIPY conjugate induced by PeT with aminoacids on the surface of a Halo‐protein,^[78b]^ and were able to design a superior non‐photobleaching rotor dye instead.^[31]^ We expect that advances in bio‐orthogonal chemistries and computational modelling of protein architecture and sensor‐protein interactions will promote the use of molecular rotors in this field.

### 3.4 Measuring nucleic acid secondary structure using molecular rotors

All the applications of molecular rotors described so far relied on conformationally‐flexible dyes reporting on solvent‐free volume in their immediate environment. Importantly, their function as rotors was underpinned by the absence of specific binding to the components of the measured systems, hence, reporting on dynamic micro‐viscosity and molecular crowding. However, some applications depend on specific binding of rotors to macromolecules of interest. One example is the binding of molecular rotor ThT to beta‐sheets of amyloid proteins (e.g. lysozyme and Aβ) to detect fibrillar aggregates. This strategy can also be used to identify nucleic acid—protein interactions,^[139]^ e.g. as demonstrated by *Dziuba* et al. who utilised a nucleoside‐linked BODIPY rotor to monitor p53 protein binding to DNA (Figure 5g).^[131]^

Similar working principles have been exploited to use environmentally sensitive probes to study base mismatches^[140]^ and non‐canonical secondary structure of nucleic acids,^[139a]^ including triplexes,^[141]^ i‐motifs^[142]^ or G‐quadruplexes (G4s).[^130^, ^143^] G4s are non‐canonical nucleic acid structures that form in guanine‐rich regions of the genome, which are thought to have vital biological roles, acting as regulators of gene transcription, in replication and in telomere maintenance.^[144]^ However, these are rare and transient structures, and developing molecular tools capable of dynamically visualising G4s is extremely important.

In our group, we employed DAOTA‐M_2_—a triangulenium‐based molecular rotor, Figure 5d—to detect G4s. This dye displays an increased fluorescence lifetime and intensity when bound to nucleic acids, due to the strong dependence of the intramolecular PeT quenching process on the conformation of the dye. PeT is alleviated in the environments of high viscosity and upon binding to DNA and RNA. We verified that DAOTA‐M_2_ binds equally strongly to duplex and G4 DNA, causing a comparable increase in fluorescence intensity. Uniquely, the lifetime observed upon binding of the dye to duplex DNA is significantly shorter compared to that bound to G4 DNA/RNA, Figure 5e[^143^, ^145^] These characteristic lifetimes are not affected by molecular crowding (e.g. when measured in DNA‐free cell extract), suggesting a prevalent effect of strong DNA binding over viscosity/crowding.^[130]^ FLIM of DAOTA‐M_2_ in live cells allowed us to establish that the level of binding to G4s can be altered by incubation with competitive (stronger) non‐fluorescent binders, such as pyridostatin or Ni‐salphen, Figure 5f.^[130]^ We proposed that the binding strength of non‐ fluorescent G4 binders (that otherwise are hard to study in cells) could be monitored through a “Fluorescence Lifetime Indicator Displacement Assay”, FLIDA, by tracking the changes of fluorescence lifetime from DAOTA‐M_2_, upon competitive displacement.^[130]^ We further utilised FLIM to monitor how some helicases (RTEL, FancJ) can unwind G4s at telomeres.^[130]^

Thiophene‐based rotors have also been explored as G4 sensors,^[146]^ and other G4 lifetime‐based probes have been reported,^[147]^ however, their sensitivity to viscosity has not been investigated. Further development of molecular rotor‐based FLIM and/or phosphorescence‐based PLIM^[148]^ probes for cellular G4s is currently ongoing in our group.

## 4 Conclusions and Perspectives

This review attempts to showcase various uses of molecular rotor fluorophores as versatile tools in quantitative biology, with applications ranging from viscosity and diffusion in lipid bilayers and plasma membranes of live cells, to temperature, and to aggregating amyloid proteins and detecting DNA secondary structure. We demonstrate that with appropriate controls these measurements have the potential to provide precise quantitative, spatially resolved and dynamic measurements of many important biological parameters.

Over the past decade biophysical approaches to understand biological processes and engineer novel diagnostic strategies are gaining traction. We believe that molecular rotor‐based approaches have much to offer in this regard, as they are capable of providing quantitative and dynamic data in natural cellular environment. One current area of great interest is the development of targetable organelle‐specific rotor probes that can provide disease‐specific information,[^31^, ^78b^, ^80c^, ^109^] while membrane‐bound rotor probes^[82]^ can open new avenues in mechanobiology research. For both the above applications brighter and more (photo)stable probes suitable for super‐resolution microscopy will be of great advantage. However, probe development is a laborious and error‐prone process. We envision that high‐throughput approaches to synthesis and testing of such probes will be key for further selection of brighter markers, and probes with fewer measurement artefacts due to non‐specific responses. In addition, we envision that computational chemistry may play a crucial role in creating vast libraries of small environmentally‐sensitive probes optimised for a breath of applications (e.g. temperature, polarity, or viscosity sensitivity). Lastly, developments in the molecular modelling approaches[^109^, ^120^, ^149^] e.g. for modelling lipid membrane dynamics or protein protein interactions, will enable expanding the use of rotors to previously unexplored areas of research.

## Conflict of interest

The authors declare no conflict of interest.

5

## Biographical Information

*Dr Miguel Paez‐Perez is a bioengineer by training (University Carlos III, Madrid) and he obtained his PhD from Imperial College London in 2022. He is currently an EPSRC Doctoral Prize Fellow in the Department of Chemistry at Imperial College London. His work sits at the interface between membrane biophysics and DNA nanotechnology, with the goal of developing novel diagnostic and therapeutic tools*.

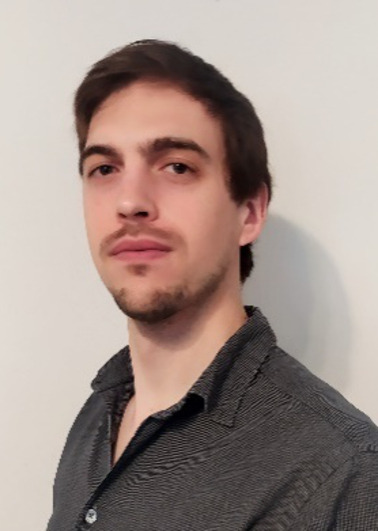

## Biographical Information

*Prof Marina Kuimova received an M.Sc. in Chemistry from Moscow State University in 2001, and a Ph.D. from Nottingham University in 2005. Following a postdoc with Prof David Phillips and an EPSRC Life Science and Career Acceleration Fellowships, held at Imperial, she was appointed as a Lecturer in 2012 and was promoted to an Associate Professor in 2016. She is currently a Professor in the Department of Chemistry. Her research interests include the elucidation of biologically relevant processes using different types of fluorescence imaging and time‐resolved spectroscopy*.

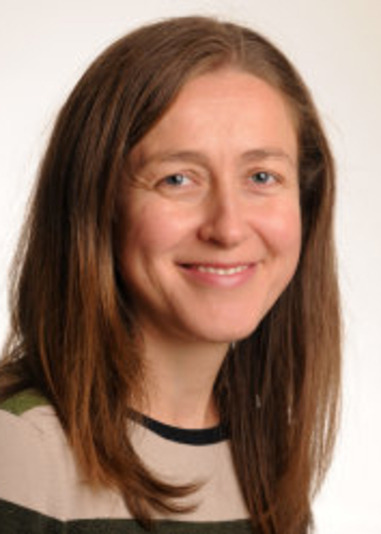

## Data Availability

Data sharing is not applicable to this article as no new data were created or analyzed in this study.
